# PlantSR: Super-Resolution Improves Object Detection in Plant Images

**DOI:** 10.3390/jimaging10060137

**Published:** 2024-06-06

**Authors:** Tianyou Jiang, Qun Yu, Yang Zhong, Mingshun Shao

**Affiliations:** 1 College of Information Science and Engineering, Shandong Agricultural University, Tai’an 271018, China; s1729041183@gmail.com (T.J.); inawjsyg@gmail.com (Y.Z.); sms1360169864@gmail.com (M.S.); 2 Huanghuaihai Key Laboratory of Smart Agricultural Technology, Ministry of Agriculture and Rural Affairs, Tai’an 271018, China

**Keywords:** super resolution, object detection, image reconstruction, image processing, deep learning, plant phenotypes

## Abstract

Recent advancements in computer vision, especially deep learning models, have shown considerable promise in tasks related to plant image object detection. However, the efficiency of these deep learning models heavily relies on input image quality, with low-resolution images significantly hindering model performance. Therefore, reconstructing high-quality images through specific techniques will help extract features from plant images, thus improving model performance. In this study, we explored the value of super-resolution technology for improving object detection model performance on plant images. Firstly, we built a comprehensive dataset comprising 1030 high-resolution plant images, named the PlantSR dataset. Subsequently, we developed a super-resolution model using the PlantSR dataset and benchmarked it against several state-of-the-art models designed for general image super-resolution tasks. Our proposed model demonstrated superior performance on the PlantSR dataset, indicating its efficacy in enhancing the super-resolution of plant images. Furthermore, we explored the effect of super-resolution on two specific object detection tasks: apple counting and soybean seed counting. By incorporating super-resolution as a pre-processing step, we observed a significant reduction in mean absolute error. Specifically, with the YOLOv7 model employed for apple counting, the mean absolute error decreased from 13.085 to 5.71. Similarly, with the P2PNet-Soy model utilized for soybean seed counting, the mean absolute error decreased from 19.159 to 15.085. These findings underscore the substantial potential of super-resolution technology in improving the performance of object detection models for accurately detecting and counting specific plants from images. The source codes and associated datasets related to this study are available at Github.

## 1. Introduction

Plant images play an important role in understanding the intricate structures and characteristics of various plant species [1,2] and are often used for automatic analysis and to extract useful information. However, images of plants often suffer from low resolution and blurriness. Even in cases where the overall image possesses high resolution, certain objects within the image may exhibit low-resolution characteristics. These limitations in image resolution and quality impede the accurate extraction of features from images, consequently restricting the performance of deep learning models created for plant image object detection tasks.

Recent advancements in computer vision, particularly deep learning models, have garnered significant attention and shown promising results in plant image object detection tasks, such as soybean seed counting [3,4] and wheat head detection [5,6,7]. Despite these successes, the effectiveness of these deep learning models heavily depends on the quality of input images [8]. Alterations in image resolution notably affect model accuracy, with low-resolution images impairing model performance during both training and inference phases [9,10,11]. Specifically, low-resolution images offer less informative content during the training phase compared to high-resolution counterparts, potentially undermining model effectiveness. Moreover, during the inference phase, images with lower resolution than training data due to capturing targets at longer distances or camera resolution limitations can also diminish model performance.

Super resolution (SR) is an image processing technique aimed at enhancing spatial resolution by reconstructing high-resolution details from low-resolution images. Deep learning-based methods have emerged as the most effective solution in the field of super-resolution [12,13,14,15]. By reconstructing high-resolution details of plant images, super-resolution technology holds the potential to empower deep learning models to effectively learn and extract intricate features from images during the training phase and improve model performance when predicting with low-resolution images during the inference phase.

Several studies have successfully applied SR techniques to plant images, enhancing the performance of deep learning models in specific tasks. For instance, Yamamoto K et al. [16] deployed the super-resolution of plant disease images for the acceleration of image-based phenotyping and vigor diagnosis in agriculture. Maqsood M H et al. [17] applied super-resolution generative adversarial networks [18] for upsampling images before using them to train deep learning models for the detection of wheat yellow rust. Cap Q H et al. [19] proposed an effective super-resolution method called LASSR for plant disease diagnosis. Albert P et al. [20] transferred knowledge learned on ground-level images to raw drone images and estimated dry herbage biomass by applying super-resolution technology to raw drone images. These successes demonstrate the significant application value of super resolution in plant image-related tasks.

While numerous datasets and models already exist in the classical field of SR [21,22,23,24], specialized SR datasets and models tailored to plant image reconstruction remain absent. In this study, we built a dedicated SR dataset solely comprising plant images (PlantSR dataset), which served as a fundamental dataset for comparing the performance of super-resolution models on plant images. Leveraging the PlantSR dataset, we compared the performance of five established deep learning models (SRCNN, VDSR, EDSR, RCAN, and SwinIR) in the classical field of SR and introduced a novel SR architecture specifically tailored to plant images (PlantSR Model). Our experimental results highlight that our model achieved the highest peak signal-to-noise ratio (PSNR) across all scaling factors (2, 3, and 4) on the PlantSR dataset while maintaining a reasonable model size and inference speed.

Next, we explored the improvement effects of our super-resolution model on apple counting and soybean seed counting tasks. In the apple counting task, applying PlantSR to test images significantly reduced the mean absolute error from 13.085 to 5.71. Similarly, for soybean seed counting, using PlantSR for both training and test images lowered the mean absolute error from 19.159 to 15.085. These findings underscore the potential of super-resolution technology to improve the performance of deep learning models for plant image-related tasks.

## 2. Materials and Methods

### 2.1. PlantSR Dataset

**Figure 1:**
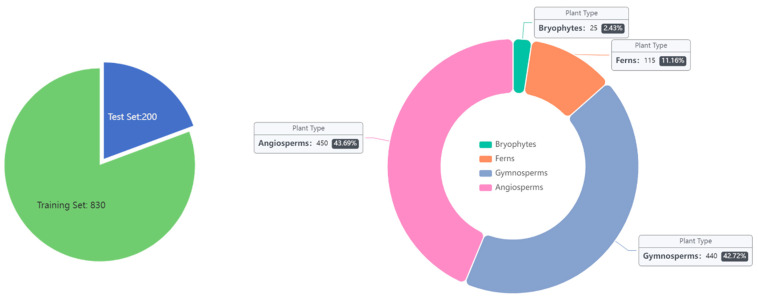
PlantSR dataset. The PlantSR dataset contains 25 images of Bryophytes, 115 images of Ferns, 440 images of Gymnosperms, and 450 images of Angiosperms. These data were divided into 830 images as a training dataset and 200 images as a test dataset.

**Figure 2:**
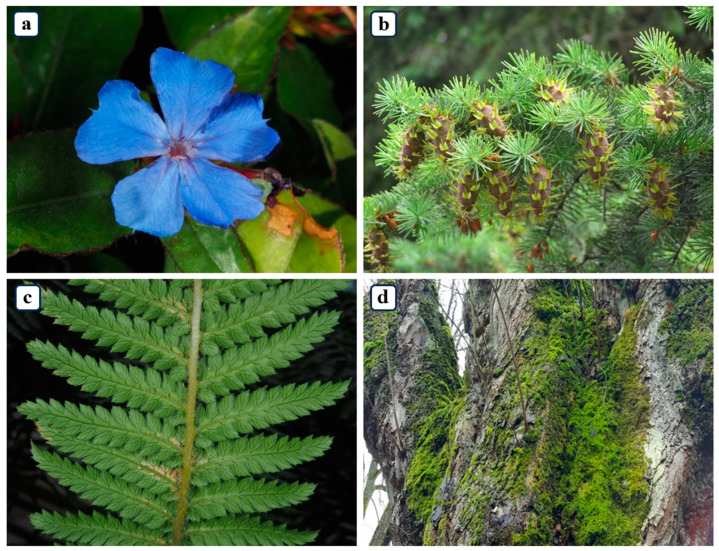
Sample images of four categories in the PlantSR dataset. (**a**) Sample image of Angiosperms. (**b**) Sample image of Gymnosperms. (**c**) Sample image of Ferns. (**d**) Sample image of Bryophytes.

The PlantSR dataset (Figure 1) comprises a total of 1030 plant images, categorized into four primary botanical groups: 25 images of Bryophytes [25], 115 images of Ferns [26], 440 images of Gymnosperms [27], and 450 images of Angiosperms [28]. The uneven distribution of plant images in the dataset reflects the biological reality of plant species diversity in nature. Being consistent with biological taxonomy, Angiosperms and Gymnosperms are typically more abundant in natural ecosystems compared to Ferns and Bryophytes. Therefore, the intentional allocation of a larger number of images to Angiosperms and Gymnosperms in the dataset is justified by their biological prevalence and economic and ecological importance. All the images were meticulously selected to ensure high resolution and sourced from Plants of the World Online [29]. These images were meticulously divided, with 830 images designated for the training dataset and 200 images for the test dataset. This dataset could be used as a fundamental dataset to compare the performance of super-resolution models on plant images in future studies. Sample images from four categories in the dataset are shown in Figure 2.

### 2.2. Architecture of PlantSR Model

**Figure 3:**
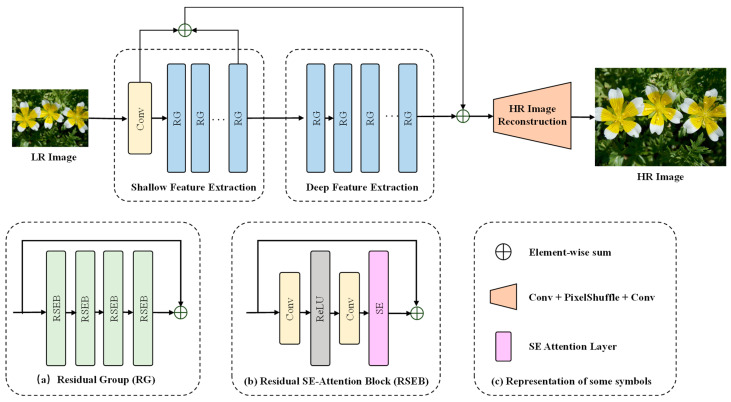
Architecture of PlantSR model.

As shown in Figure 3, our PlantSR model consisted of three modules: shallow feature extraction, deep feature extraction, and high-resolution (HR) image reconstruction.

Given a low-resolution (LR) image *I_LR_* as input, we first used a 3 × 3 convolutional layer to extract feature $F_{0}$

(1) $$F_{0}=H_{SF\_0}\left(I_{LR}\right),$$

where *H_SF_*__0_ denotes a 3 × 3 convolutional layer, which was defined as the first component of the shallow feature extraction module. Then, we used the second component of the shallow feature extraction module to extract shallow feature $F_{1}$ from $F_{0}$, and used the deep feature extraction module to extract deep feature $F_{DF}$ from $F_{1}$

(2) $$F_{1}=H_{SF\_1}\left(F_{0}\right),$$

(3) $$F_{DF}=H_{DF}\left(F_{1}\right),$$

where *H_SF_*__1_ denotes the second component of the shallow feature extraction module and *H_DF_* (·) denotes the deep feature extraction module; these two modules consisted of m and n residual groups (RGs), respectively. Each RG was composed of four Residual SE-attention Blocks (RSEBs) and a residual connection from input to output features. Within each RSEB, an SE-attention layer [30] was applied sequentially after a convolutional layer, a rectified linear unit (ReLU) layer, and another convolutional layer. Once feature maps were obtained from the deep feature extraction module, the HR image reconstruction module was performed to obtain the SR image:

(4) $$I_{SR}=H_{UP}\left(F_{0}+F_{1}+F_{DF}\right),$$

where *H_UP_* (·) denotes the HR image reconstruction module, which was initiated by enhancing the feature map channels through a convolutional layer. Following this, the module scaled up the feature maps using a PixelShuffle layer and, in the concluding step, reconstructed the feature maps to the RGB channel through another convolutional layer.

Throughout the training phase, we optimized the parameters by minimizing the L1 pixel loss:

(5) $$L=||I_{SR}-I_{HR}|{|}_{1},$$

where $I_{SR}$ is obtained by taking LR image $I_{LR}$ as the input of PlantSR and $I_{HR}$ is the corresponding ground-truth HR image. We opted for the L1 loss, as it has demonstrated superior performance in image restoration tasks [31]. The loss function was optimized using the Adam optimization algorithm.

### 2.3. Super-Resolution Effects on Apple Counting Task

As YOLOv7 is one of the most popular models in the field of object detection, we selected it to analyze our super-resolution model effects on counting apples [32]. For training and evaluating the apple counting model, we sourced 1000 images from the internet to form the training dataset. Additionally, we utilized 200 images of harvest-ready apples provided by the 2023 Asia and Pacific Mathematical Contest in Modeling competition as our test dataset. Each image in the test dataset was relatively small, all measuring 270 × 180 pixels.

After training the YOLOv7 model using 1000 images in the training dataset, we conducted tests with and without applying super-resolution to the test images. Super-resolution was achieved using the PlantSR (×3) model. To ensure optimal performance in processing apple images, we fine-tuned a PlantSR model using a curated collection of 100 high-resolution close-up apple images gathered from the internet, leveraging these images to train a model based on the original pretrained model.

### 2.4. Super-Resolution Effects on Soybean Seed Counting Task

As an improvement to the P2PNet [33], Zhao J et al. proposed an automated soybean seed counting tool called P2PNet-Soy [34]. Thanks to their efforts, the accuracy of soybean seed counting in the field witnessed a significant improvement. P2PNet-Soy incorporates an unsupervised clustering algorithm, the k-d tree [35], as post-processing to find the centers of closely located predictions, thereby enhancing the final prediction accuracy. Additionally, P2PNet-Soy made some adjustments to the architecture of P2PNet to maximize model performance in seed counting and localization. We downloaded their open dataset, which consists of 126 images of soybeans captured from one side as the training dataset and 132 images of soybeans taken from the opposite side as the evaluation dataset [36].

Expanding on the open dataset, we incorporated 60 soybean seed images from SDAU Technology Park and reorganized the dataset as follows: 126 images from the P2PNet-Soy training dataset plus 30 additional images as the training dataset, 50 images from the P2PNet-Soy evaluation dataset plus 10 additional images as the validation dataset, and 82 images from the P2PNet-Soy evaluation dataset as the test dataset.

To explore the effects of super-resolution on the task of soybean seed counting, we trained and evaluated P2PNet-Soy with images in four different situations: (1) Original images across training, validation, and test sets. All images used for training, validation, and testing purposes remained in their original resolution. (2) Original training and validation images with downsampling of test images. The training and validation images remained in their original resolution, while the test images underwent downsampling by a factor of two using bilinear interpolation. (3) Original training and validation images with downsampling and PlantSR (×2) upscaling of test images. Similar to the previous setup, the training and validation images remained in their original resolutions. However, for the test set, images underwent downsampling followed by upscaling using a PlantSR (×2) model. (4) PlantSR (×2) upscaled images across training, validation, and test sets. All images used for training, validation, and testing underwent upscaling by a PlantSR (×2) model.

**Figure 4:**
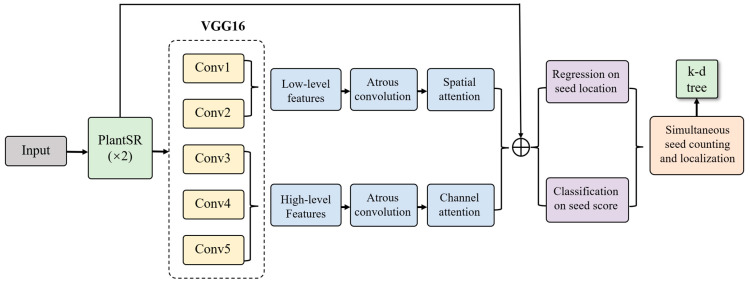
Architecture of P2PNet-Soy after applying the PlantSR model as a pre-processing step.

In the fourth case, we proposed the application of the PlantSR model as a pre-processing step to P2PNet-Soy (Figure 4). Here is a detailed description of this setup: Before employing images for training, validation, and testing in P2PNet-Soy, all images underwent upscaling by a PlantSR (×2) model. To train a specific PlantSR model on soybean seed images, we collected 89 high-resolution close-up images of soybeans from laboratory settings. These images, possessing a higher pixel density than those in the original dataset, were used for transfer learning to train the specific PlantSR (×2) model on soybean seed images. During the training phase, P2PNet-Soy randomly cropped 224 × 224 patches from the original images. Considering the upscale by a factor of two, the resolution of these patches was adjusted to 448 × 448. Moreover, P2PNet-Soy used a k-d tree to filter the prediction results. In the implementation of the k-d tree, the parameter cutoff was set to find all points within the distance “r” from each point. Since the pixel density of the images was tripled, the parameter cutoff was also doubled in our experiment.

### 2.5. Training and Evaluation Settings

To assess the performance of various super-resolution (SR) models, including our PlantSR model, consistent training and evaluation settings were applied. All models were trained and evaluated on the PlantSR dataset and shared identical configurations for training. In each training batch, 32 patches sized 64 × 64 (for scale = 2 and scale = 4) or 63 × 63 (for scale = 3) were extracted from training images, followed by horizontal data augmentation. A total of 38,997 batches constituted one epoch. Each model was trained for multiple epochs, and their evaluation metrics on the test dataset were recorded. The model that performed best in multiple epochs was saved for final comparison. We used Adam [37] as the optimizer and the initial learning rate was set to 10^−4^. All the SR models were trained on an NVIDIA RTX A5000 24G GPU (NVIDIA, Santa Clara, California, USA).

To explore the effects of the PlantSR model on the task of apple counting, we trained and evaluated YOLOv7 with test images with or without applying super-resolution to the test images. The input size of YOLOv7 was set to 640. A series of data augmentations (mosaic, mixup, random crop, random angle) were applied throughout the training phase. The training of YOLOv7 was conducted on an NVIDIA RTX A5000 24G GPU.

To explore the effects of the PlantSR model on the task of soybean seed counting, we trained and evaluated P2PNet-Soy with images in four different cases. In all four cases, a series of data augmentations (random scale, random crop, random flipping, random change brightness) were applied throughout the training phase, mirroring the approach used for the original P2PNet-Soy. We used Adam as the optimizer and the initial leaning rate was set to 10^−4^ and subsequently halved every 100 epochs. The training of P2PNet-Soy was conducted on an NVIDIA A100 40G Tensor Core GPU (NVIDIA, Santa Clara, CA, USA).

### 2.6. Evaluation Metrics

To compare the performance of several SR methods, we used two common metrics of image quality, including peak-to-peak signal-to-noise ratio (PSNR) and the structural similarity index measure (SSIM) [38]. The higher the PSNR and SSIM, the higher the similarity between predicted images and high-resolution images, and the better the model performance. The computation of these metrics was as follows:

(6) $$PSNR=10\cdot \log_{10}\left(\frac{MAX^{2}}{MSE}\right),$$

where *MAX* represents the maximum possible pixel value in the image, which was 255 here. *MSE* denotes the mean squared error, indicating the average of squared differences between corresponding pixels in the original and predicted images:

(7) $$SSIM=\frac{\left(2\mu_{x}\;\mu_{y}\;+C_{1}\;)(2\sigma_{xy}\;+C_{2}\;\right)}{\left({\mu_{x}}^{2}\;+{\mu_{y}}^{2}\;+C_{1}\;)({\sigma_{x}}^{2}\;+{\sigma_{y}}^{2}\;+C_{2}\;\right)},$$

where $\mu_{x}$ and $\mu_{y}$ are the means of images x and y, respectively. ${\sigma_{x}}^{2}$ and ${\sigma_{y}}^{2}$ are the variances of images x and y, respectively. $\sigma_{xy}$ represents the covariance between x and y. $C_{1}$ and $C_{2}$ equal ${\left(0.01L\right)}^{2}$ and ${\left(0.03L\right)}^{2}$, where L is the dynamic range of pixel values, which was 255 here.

We used mean absolute error (MAE) and root mean square error (RMSE) to evaluate the performance of the soybean seed counting model and apple counting model. The closer the *MAE* and *RMSE* were to 0, the smaller the error in counting:

(8) $$MAE=\;\frac{1}{n}\sum_{i=1}^{n}\left|{\hat{y}}_{i}-y_{i}\right|$$

(9) $$RMSE=\sqrt{\frac{1}{n}\sum_{i=1}^{n}{\left({\hat{y}}_{i}-y_{i}\right)}^{2}}$$

where *n* represents the number of images used for testing, $y_{i}$ represents the ground truth number of the target object within each image, and ${\hat{y}}_{i}$ represents the number of target objects predicted by the model.

## 3. Results

Our experimental results are presented in three sections: (1) SR model compression. Based on the PlantSR dataset, we conduct benchmarking of our PlantSR model against five well-established super-resolution (SR) models in the classic field of SR, alongside a traditional method, bicubic interpolation. (2) Super-resolution effects on the apple counting task. We trained and evaluated the YOLOv7 model with test images with or without applying the PlantSR model to achieve super resolution. (3) Super-resolution effects on the soybean seed counting task. We delved into the impact of our PlantSR model on the soybean seed counting task. Here, we meticulously trained and evaluated the P2PNet-Soy model with images in four different cases.

### 3.1. SR Model Compression

We rigorously benchmarked our proposed PlantSR network against a traditional method, bicubic interpolation, and five state-of-the-art super-resolution (SR) deep learning models: SRCNN [39], VDSR [40], EDSR [41], RCAN [42], and SwinIR [43]. Among the five SR deep learning models, SRCNN and VDSR employed interpolation to the low-resolution (LR) image as a preliminary step, subsequently learning to reconstruct the high-resolution (HR) image from these interpolated images. This approach is commonly referred to as Pre-upsampling SR. Conversely, EDSR, RCAN, and SwinIR first extracted features from LR images and then applied the upsampling module at the end of the network. This methodology is commonly referred to as Post-upsampling SR. Notably, our PlantSR model also belonged to the Post-upsampling SR category.

Table 1 presents the quantitative results, where the number of parameters is expressed in millions, frames per second (FPS) are denoted in frames, and the input size is specified in the number of pixels. During the training process, patches of sizes 64 × 64 (for scale = 2 and scale = 4) or 63 × 63 (for scale = 3) were utilized as high-resolution (HR) images and were subsequently downsampled by a factor of scale as low-resolution (LR) images. The models learned the transformation process from LR to HR. It is noteworthy that the reported FPS results, although averaged across multiple experimental outcomes, should be considered as reference points due to inherent variations.

**Figure 5:**
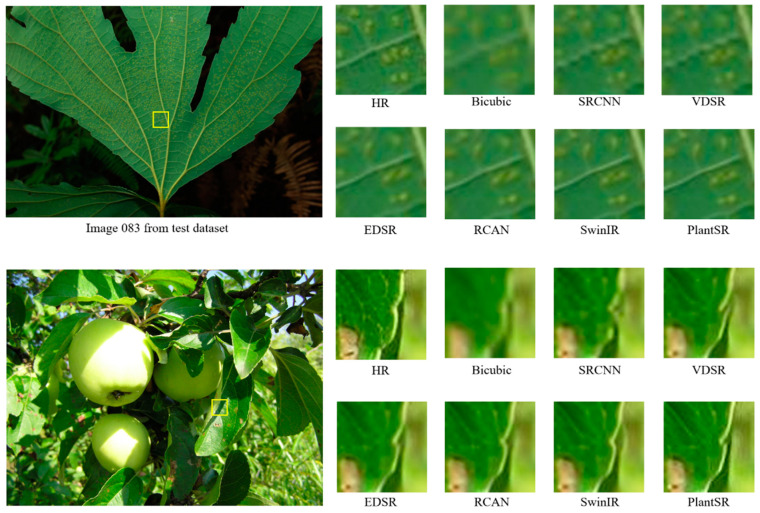
Visual comparison results of image SR (×4) methods.

The results demonstrate that our PlantSR model achieved exceptional SR performance on plant images while maintaining a reasonable model size and inference speed. Among the compared models, it was observed that SwinIR, which uses Swin Transformer [44] as its backbone network, exhibited satisfactory performance. However, it exhibited comparatively slower inference speeds and higher computational expenses. Visual comparison results of SR (×4) are illustrated in Figure 5; the patches for comparison are marked with yellow boxes in the original images (left).

### 3.2. Super-Resolution Effects on the Apple Counting Task

We trained a YOLOv7 model using 1000 images collected from the internet and evaluated the model on test images with or without using PlantSR (×3) to achieve super resolution. Before evaluation, we collected some high-resolution close-up images of apples to train a specialized PlantSR (×3) model that had superior performance in upscaling apple images. We used the PlantSR (×3) model pretrained on the PlantSR dataset as the initialization model and conducted further training on these high-resolution apple images, thereby obtaining a specialized PlantSR (×3) model for processing apple images. Before applying super resolution to test images, the resolution of these images was 270 in width and 185 in height. After applying the PlantSR (×3) model, the resolution of these images increased to 810 (270 × 3) in width and 555 (185 × 3) in height. Next, we tested the YOLOv7 model separately under these two cases of test images.

**Figure 6:**
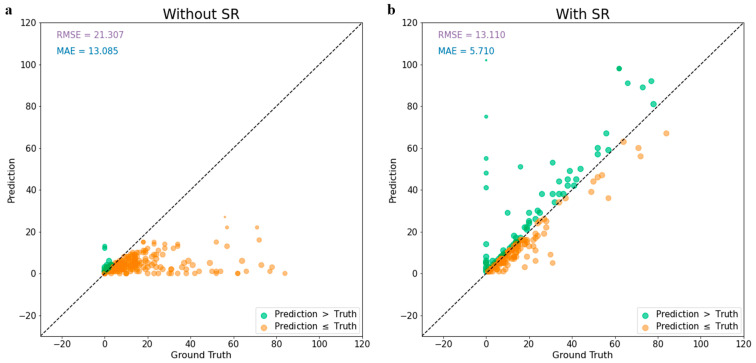
Test results of YOLOv7 with different cases of test images. (**a**) test images without SR. (**b**) test images with SR.

Before applying the PlantSR model to achieve super resolution, the mean absolute error of the apple counting model on test images was 13.085 (Figure 6a). Notably, a significant portion of the images exhibited an under-detection of apples, with predicted values falling below the ground truth values. However, post-application of super resolution to the test images, the mean absolute error notably decreased to 5.71, which showed a great improvement in the model performance (Figure 6b).

### 3.3. Super-Resolution Effects on the Soybean Seed Counting Task

**Figure 7:**
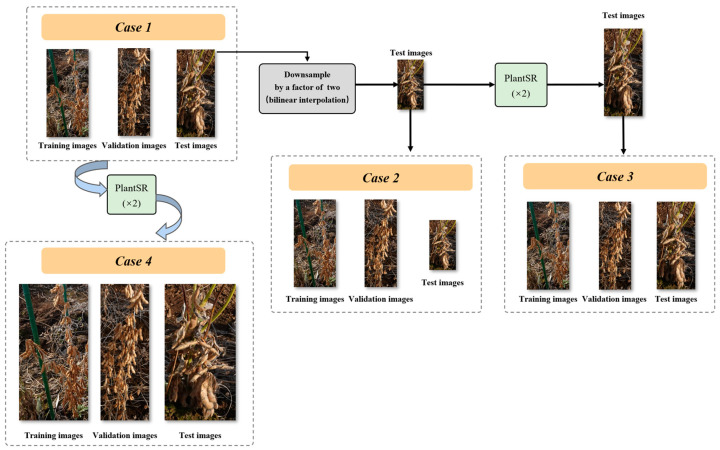
Training, validation, and test images of P2PNet-Soy in four different cases.

We trained and evaluated P2PNet-Soy with images in four different cases (Figure 7): (1) original images across training, validation, and test sets; (2) original training and validation images with downsampling of test images; (3) original training and validation images while test images underwent downsampling followed by upscaling using a PlantSR (×2) model; and (4) PlantSR (×2) upscaled images across training, validation, and test sets.

For the third and fourth cases, we used a PlantSR (×2) model for processing images. Despite the availability of previously trained models for resolution enhancement, we aimed to train a specialized PlantSR (×2) model with superior performance in upscaling these soybean seed images. To achieve this, we curated a collection of high-resolution close-up images of soybeans from laboratory settings, characterized by a higher pixel density compared to the soybean seed images used for training and evaluating P2PNet-Soy. We used the PlantSR (×2) model pretrained on the PlantSR dataset as the initialization model and conducted further training on these high-resolution soybean images, obtaining a specialized PlantSR (×2) model for processing soybean seed images.

Table 2 shows the test results of P2PNet-Soy with different cases of images. Comparing the case where all images remained in their original resolution (Case 1) with the case where all images were upscaled using the PlantSR (×2) model (Case 4), we observed a reduction in MAE from 19.16 to 15.09. However, when evaluating the model trained on original images using downscaled test images (Case 2), the MAE increased significantly to 59.23. Notably, implementing the PlantSR (×2) model for these downscaled test images (Case 3) led to a noteworthy reduction in MAE, successfully minimizing it to 19.82, a value close to that achieved in Case 1.

## 4. Discussion

Plant images are often used for automatic analysis and to extract useful information. Due to some limitations, the objects in these images may suffer from low resolution. For instance, when counting soybean seeds through deep neural networks, we usually take images that include the entire soybean plant. Although the entire image is in high resolution, the soybean seeds in the images can still be in low resolution. Another instance is that when using drones to capture images, it is difficult to obtain high-resolution images of the targets on the ground because of the flying altitude of the drone, which results in a longer shooting distance. These limitations in object resolution may lead to insufficient information, resulting in a decrease in the performance of computer vision models.

The foundation of our approach rested on the creation of the PlantSR dataset, comprised exclusively of high-resolution plant images. This dataset not only facilitated the evaluation of SR models but also served as a valuable resource for benchmarking their performance. By ensuring the quality and diversity of images in this dataset, we aimed to provide a robust platform for comparing various SR methodologies.

Through extensive experimentation, we built a PlantSR model with the most effective solution for restoring high-resolution plant images from their low-resolution counterparts. Notably, we deliberately avoided transformer-based architectures in favor of alternative methodologies optimized for efficient inference in plant imagery [45,46]. This decision was driven by the need for practical inference speed and compelling performance gains, as evidenced by achieving the highest peak signal-to-noise ratio (PSNR) across all scaling factors (2, 3, and 4) on the PlantSR dataset.

Furthermore, we conducted experiments to explore the effects of super-resolution on two object detection tasks: apple counting and soybean seed counting. Our results demonstrate significant improvements in counting performance when applying the PlantSR model, particularly when images are initially in low resolution or downsampled. In the soybean seed counting task, compared with the case where all images remained in their original resolution, applying the PlantSR (×2) model to upscale all images successfully reduced the MAE of P2PNet-Soy from 19.16 to 15.09. This enhancement in counting performance can be attributed to several factors. Firstly, the increased pixel density of the soybean seed images facilitated by the SR model enabled the counting model to discern and learn more intricate features. Secondly, by processing all images using the same SR model, a higher level of similarity among the images was achieved, potentially adapting the domain between the training and test images [47]. However, it was observed that downsampling test images led to a substantial decline in model performance, underlining the significant influence of image resolution on model performance. Notably, implementing the PlantSR (×2) model for these downsampled test images resulted in a remarkable reduction in MAE, minimizing it from 59.23 to 19.89. The apple counting task obtained similar results, and applying the PlantSR (×3) model to the low-resolution test images decreased the MAE from 13.085 to 5.71.

Besides these successes, certain limitations are inherent in our current methodology. Firstly, the PlantSR model, tailored specifically to super-resolution plant images, might not perform optimally for some other tasks in image reconstruction such as artifact reduction and denoising. Secondly, our focus was predominantly on the application of super resolution to RGB images. Nonetheless, beyond RGB images, some other data types captured by various sensors exist, including images from multiple layers of channels taken by multispectral cameras, depth maps obtained by depth cameras, and three-dimensional point clouds acquired through lidar scanning. Super resolution for these diverse data types remains an invaluable and challenging endeavor. Thirdly, enhancing the resolution of input images is likely to result in a slowdown in both the training and inference speed of deep learning models. This is particularly notable given the increased computational cost during the training phase.

In conclusion, our study underscores the potential of super-resolution technology to improve the performance of object detection models in plant image analysis. The development of specialized SR models, exemplified by the PlantSR model, holds immense promise for restoring high-resolution plant images and thereby enhancing the capabilities of computer vision models reliant on such images. Future research directions may involve delving into a generalized model capable of addressing multiple subtasks of image reconstruction for plant images, proposing more extensive models capable of handling a broader range of data types.

## Figures and Tables

**Table 1:** Compression results of SR methods.

Method	Scale	PSNR	SSIM	Params (M)	FPS	Input Size (Pixels)
Bicubic	×2	38.23	0.9617	/	/	64
SRCNN		39.42	0.9667	0.069	507.0	
VDSR		40.31	0.9716	0.667	231.3	
EDSR		40.26	0.9706	1.370	123.7	
RCAN		40.15	0.9708	15.445	6.6	
SwinIR		40.34	0.9715	16.619	0.8	
PlantSR (Ours)		40.36	0.9716	1.397	37.1	
Bicubic	×3	33.56	0.9128	/	/	63
SRCNN		34.24	0.9190	0.069	475.4	
VDSR		34.79	0.9255	0.667	235.3	
EDSR		35.20	0.9273	1.554	71.9	
RCAN		35.24	0.9290	15.629	6.6	
SwinIR		35.23	0.9290	16.803	0.8	
PlantSR (Ours)		35.26	0.9291	5.760	18.1	
Bicubic	×4	32.13	0.8844	/	/	64
SRCNN		32.64	0.8927	0.069	473.2	
VDSR		33.53	0.9031	0.667	236.6	
EDSR		33.76	0.9063	1.518	57.5	
RCAN		33.78	0.9071	15.888	6.5	
SwinIR		33.83	0.9075	16.766	0.7	
PlantSR (Ours)		33.85	0.9077	13.531	9.8	

**Table 2:** Train and evaluate P2PNet-Soy with images in four different cases.

Case	Preprocessing	MAE	RMSE
Case 1	No	19.16	21.49
Case 2	Downsample test images	59.23	61.47
Case 3	Downsample test images and then upscale them using a PlantSR (×2) model	19.82	22.91
Case 4	Upscale all the images	15.09	22.19

## Data Availability

The source code is available at https://github.com/SkyCol/PlantSR (accessed on 28 November 2023). The PlantSR dataset is available at figshare [48]. The high-resolution soybean seed images are available at figshare [49].
